# Sex Bias in the Clinical Diagnosis of ADHD: A Critical Review

**DOI:** 10.3390/jcm15176804

**Published:** 2026-09-02

**Authors:** Farzad Salehpour, Francisco Gonzalez-Lima

**Affiliations:** 1 Department of Psychology, Texas Consortium in Behavioral Neuroscience, The University of Texas at Austin, Austin, TX 78712, USA; gonzalezlima@utexas.edu; 2 Interdisciplinary Neuroscience Program, Department of Psychiatry and Behavioral Sciences, Dell Medical School, The University of Texas at Austin, Austin, TX 78712, USA

**Keywords:** attention-deficit/hyperactivity disorder, sex bias, diagnostic bias, referral bias, DSM criteria

## Abstract

**Background:** Attention-deficit/hyperactivity disorder (ADHD) is a prevalent neurodevelopmental disorder that manifests differently across sexes, potentially impacting symptomatology, diagnosis, and treatment. Nevertheless, these legitimate sex differences in ADHD are further shaped by multiple sources of bias that distort prevalence estimates and clinical recognition. **Methods:** This critical review synthesizes evidence on a variety of factors, including sampling bias, referral bias, DSM criteria, and diagnostic overshadowing, that may contribute to the under-recognition and misdiagnosis of ADHD in females. **Results:** Studies suggest that sampling and referral biases inflate male representation by overemphasizing disruptive behaviors and underrecognizing inattentive presentations in females. Item-level symptom endorsement differences indicate that several DSM-5 criteria map more closely onto male-typical behaviors, reducing sensitivity to female-specific manifestations. Diagnostic thresholds, age-of-onset, and pervasiveness criteria further disadvantage females, whose symptoms are often less overt or more context-dependent. Recognition bias and diagnostic overshadowing by mood and anxiety disorders contribute to missed or delayed diagnoses. **Conclusions:** Together, these findings highlight that observed sex differences in ADHD reflect not only true psychobiological variation but also systematic biases in assessment and diagnosis, underscoring the need for more sex-sensitive diagnostic frameworks and improved clinical awareness. The extent of these diagnostic disparities may also vary across cultural contexts and healthcare systems, including differences in symptom perception, referral pathways, and access to specialist assessment.

## 1. Introduction

Attention-deficit/hyperactivity disorder (ADHD) is one of the most common neurodevelopmental disorders across the lifespan and is characterized by persistent symptoms of inattention, hyperactivity, and impulsivity that interfere with functioning and development. For decades, ADHD has been regarded as a predominantly male disorder, a perception largely shaped by consistently higher diagnosis rates among boys than girls. Current epidemiological evidence indicates a male-to-female ratio of approximately 4:1 in clinic-referred childhood samples [1] and between 2.4:1 [1] and 3.2:1 [2] in community-based populations. A recent large population-based twin study further demonstrated that while the male-to-female ratio for ADHD was 2.5:1 at the clinical diagnosis level, it decreased to 1.8:1 at the population level [3].

The interpretation of these sex differences remains a subject of considerable debate. One possibility is that the higher prevalence observed in males reflects genuine sex-related differences in the neurobiological and developmental mechanisms underlying ADHD. Indeed, a growing body of literature has documented sex-related differences in symptom expression, neuropsychological functioning, neurodevelopmental trajectories, endocrine influences, and genetic risk factors [4,5]. However, an alternative and increasingly influential perspective argues that at least part of the apparent male predominance may result from systematic biases operating throughout the diagnostic process [6]. From symptom recognition and referral patterns to assessment instruments and diagnostic criteria, multiple factors may contribute to the under-identification, delayed diagnosis, or misdiagnosis of females with ADHD [6,7].

Historically, the conceptualization of ADHD has been heavily influenced by research conducted predominantly in male samples. Early clinical descriptions emphasized disruptive, externally observable behaviors such as hyperactivity, impulsivity, and classroom misconduct, characteristics that are generally more common and more readily recognized in boys [8]. In contrast, females with ADHD often present with less overt manifestations, including inattentiveness, internal distractibility, emotional dysregulation, excessive daydreaming, and difficulties in social or academic functioning [9]. Because these symptoms are less disruptive and may be more easily concealed through compensatory strategies, they frequently attract less attention from parents, teachers, and healthcare professionals. Consequently, many girls remain unidentified during childhood and only receive a diagnosis later in adolescence or adulthood after experiencing substantial functional impairment [6].

Recognition of these concerns has prompted increasing scrutiny of the extent to which current diagnostic practices accurately identify ADHD across sexes [10]. Importantly, understanding diagnostic sex bias extends beyond questions of prevalence. Delayed or missed diagnosis can have substantial consequences, including reduced access to evidence-based interventions, increased academic and occupational difficulties, heightened emotional distress, and elevated risk for psychiatric comorbidities [3,9]. Clarifying the extent to which current diagnostic practices contribute to these disparities is therefore essential for improving assessment procedures and ensuring equitable clinical care across sexes [11].

A growing literature suggests that methodological, diagnostic, and sociocultural factors may contribute to the observed male predominance in ADHD diagnosis [6]. This review critically examines the evidence for diagnostic sex bias in ADHD, with particular emphasis on sampling and referral processes, symptom measurement, DSM diagnostic criteria, functional impairment, informant recognition, and diagnostic overshadowing. By evaluating these factors collectively, we aim to clarify their potential contribution to the observed male-female disparity in ADHD diagnosis and identify important directions for future investigation. It is important to distinguish biological sex, gender, and sociocultural influences, which are related but not interchangeable. Biological sex refers to biologically based male-female differences, whereas gender reflects identity, expression, roles, and socially shaped expectations [12]. Sociocultural influences encompass broader social and cultural norms and expectations that may shape how ADHD-related behaviors are expressed, perceived, and recognized. Because most ADHD studies classify participants simply as male or female without directly assessing gender-related factors, the term “sex” is used throughout this review while acknowledging that some observed differences may also reflect gender-related and sociocultural influences [13].

## 2. Literature Search and Methodological Approach

This critical narrative review aims to synthesize evidence regarding factors that may contribute to sex-related bias in the clinical recognition and diagnosis of ADHD. A structured literature search was conducted in Web of Science, PubMed, Embase, and Scopus from database inception through December 2025. The database search was also supplemented by manual screening of the reference lists of relevant articles to identify additional relevant studies not captured through the database searches.

Search terms included combinations of “attention-deficit/hyperactivity disorder”, “sex differences”, “gender differences”, “sex bias”, “gender bias”, “diagnostic bias”, “female”, “male”, “girls”, “boys”, “women”, “men”, “diagnosis”, “underdiagnosis”, “misdiagnosis”, “referral bias”, “recognition bias”, “DSM criteria”, “symptom endorsement”, “diagnostic threshold”, “age of onset”, “late-onset ADHD”, “functional impairment”, “informant agreement”, “pervasiveness”, “diagnostic overshadowing”, and related terms.

Titles and abstracts of English-language articles were screened for relevance, followed by full-text evaluation of studies considered pertinent to the objectives of the manuscript. Articles were considered relevant when they examined individuals with ADHD or elevated ADHD symptoms and provided evidence concerning sex- or gender-related differences that could influence symptom recognition, referral, assessment, the fulfillment of diagnostic criteria, or receipt of an ADHD diagnosis. Studies addressing differences in symptom expression, diagnostic thresholds, age of onset, functional impairment, informant reporting, psychiatric comorbidity, and diagnostic pathways were also considered when directly relevant to understanding potential diagnostic bias. Studies focused exclusively on biological or neuropsychological sex differences without implications for clinical recognition or diagnosis were outside the primary scope of the review.

Because the objective of this review was to provide a critical synthesis of a broad and heterogeneous literature rather than a systematic review of a narrowly defined research question, the review was not conducted according to PRISMA guidelines, and no formal quantitative synthesis or standardized risk-of-bias assessment was performed. Study selection was therefore guided by relevance to the principal sources of potential diagnostic sex bias examined in the review. Greater interpretive weight was given to clinical studies directly evaluating diagnostic or referral processes, large population-based and epidemiological studies, longitudinal studies, psychometric studies examining differential item functioning or measurement invariance, as well as systematic reviews and meta-analyses. Commentaries and expert consensus papers were also included when necessary to provide conceptual or clinical context. Where evidence was inconsistent or primarily interpretive, this was considered when evaluating the strength of the conclusions. The evidence was synthesized narratively with attention to study design, sample characteristics, developmental stage, diagnostic methods, informant source, and clinical context.

ChatGPT-5 (OpenAI) was utilized during the preparation and revision of the manuscript as a writing and editorial tool to enhance the clarity and language of the authors’ synthesis of the literature. All content generated with AI assistance was carefully reviewed, verified, and revised by the authors, who assume full responsibility for the final manuscript.

## 3. Factors That May Contribute to Sex Bias in ADHD Diagnosis

### 3.1. Sampling Bias and Sex Referral Bias

Historically, the DSM-IV field trials that established the diagnostic criteria for ADHD were conducted in a sample that included only 21% females [14]. The DSM-5 field trials likewise showed a sex imbalance, with females representing only about 40% of the pediatric participants, even though the adult sites enrolled males and females in nearly equal numbers (48% females and 52% males) [15]. For many years, experts have argued that the current DSM criteria are male-biased, making them more appropriate for the behavioral patterns typically seen in boys and less accurate for diagnosing ADHD in girls [6,16].

Another major concern is that much of our understanding of sex differences in ADHD comes from clinic-referred samples, which are subject to “sex referral bias” [17]. Sex referral bias refers to the fact that ADHD clinics receive more referrals for boys compared to girls [5]. Empirical evidence suggests that boys’ externalizing and disruptive behaviors may be more likely to prompt referral, whereas girls with primarily inattentive symptoms and fewer overt disruptions (e.g., conduct disorder) may be more likely to be overlooked [18,19]. As a result, the girls who do reach clinics tend to exhibit especially disruptive or combined-type behaviors [3,20] and therefore do not represent the majority of females with ADHD in the community, most of whom present primarily with inattentive symptoms [21]. Supporting this view, meta-analyses show that clinic-referred ADHD girls are not representative of non-referred girls in the same way that boys are [21,22]. In sum, these considerations underscore the need to examine community-based samples to obtain a more accurate picture of sex differences in ADHD.

### 3.2. Item-Level Symptom Endorsement

Studies examining the psychometric properties of ADHD rating scales have tested whether the DSM item-level symptom criteria differentiate equally well between boys and girls [23,24,25]. Evidence from longitudinal parent ratings of the 18 DSM-IV ADHD symptom items indicates that, for the most part, these items show strong measurement invariance and minimal differential item functioning across sex from preschool through ninth grade. This finding suggests that the majority of the DSM-IV-defined symptoms assess the same underlying constructs of inattention and hyperactivity/impulsivity in both boys and girls [23]. However, some studies have identified exceptions within both the inattention and hyperactivity/impulsivity symptom dimensions [26,27,28].

Parent ratings show that the items *“fidgets with hands or feet”*, *“leaves seat in classroom”*, and *“runs about or climbs excessively”* are more likely to be endorsed for boys than for girls, whereas *“talks excessively”* and *“interrupts others”* are more likely to be endorsed for girls than for boys at the same latent hyperactivity/impulsivity trait level. Teacher ratings reveal a similar pattern in which “*fidgets with hands or feet*” is more often endorsed for boys and “*talks excessively*” for girls. Overall, these findings indicate that sex differences were present in hyperactivity/impulsivity items but not in inattention symptom items [28]. These findings were further extended by a recent meta-analysis demonstrating sex differences across both hyperactivity/impulsivity and inattention symptom items. Williams et al. found that parent-rated data on children and adolescents with ADHD showed medium-to-high endorsement rates for symptoms with significant sex differences. Specifically, parents reported that females were more likely than males to exhibit the symptoms *“easily distracted”* (odds ratio [OR] = 1.54) and “*fails to sustain attention in tasks*” (OR = 1.39). In contrast, males were more frequently described as displaying *“blurts out answers”* (OR = 0.86), *“interrupts others”* (OR = 0.75), and *“talks excessively”* (OR = 0.93). The strongest effects were observed for *“easily distracted”* and *“blurts out answers”.* No significant sex differences were found for the remaining eight items. Similarly, self-reported data from adults with ADHD indicated high endorsement rates for symptoms showing significant sex differences. Adult females were more likely than males to endorse *“often easily distracted”* (OR = 1.49), *“often has difficulty organizing tasks”* (OR = 1.40), *“often blurts out answers”* (OR = 1.32), and *“often talks excessively”* (OR = 1.65). The largest sex differences were observed for *“often easily distracted”* and *“often talks excessively,”* with females more likely to endorse these symptoms, consistent with other endorsement-based findings [25]. No significant sex differences were found for the remaining 12 items [26].

The findings from Williams et al. also showed that, for hyperactivity/impulsivity symptoms in children and adolescents, boys were more likely to display eight of the nine DSM-IV hyperactive/impulsive symptoms, with *“talks excessively”* being the only exception. This raises important questions about how ADHD symptoms manifest and are captured across sexes. Consistent with these findings, empirical studies have demonstrated that some hyperactivity/impulsivity symptoms may be expressed or perceived differently across sexes, with certain DSM-based symptom items potentially reflecting behaviors perceived as more male-typical [27,28]. These findings raise concerns that the items *“fidgets with hands or feet”, “leaves seat in classroom”, “runs about or climbs excessively”, “talks excessively”,* and *“interrupts others”* may not capture ADHD behaviors equally well for children and adolescents of different sexes. To address this issue, researchers have suggested collecting qualitative data through interviews with separate samples of parents and teachers of boys and girls with ADHD to determine which behaviors best describe hyperactivity and impulsivity in each group. By identifying behavior descriptions that are commonly reported regardless of a child’s sex, rather than those unique to boys or girls, symptom items could be refined to reduce sex-related differential item functioning and improve sensitivity to female presentations [28]. It has also been suggested that the nature of hyperactivity/impulsivity behaviors in females may be more oriented toward social relationships, in contrast to the more task-focused behaviors emphasized in current diagnostic criteria [27,29]. Therefore, incorporating interpersonal behaviors such as impulsively changing conversation topics or being forgetful or late for social activities could better capture female-specific manifestations of ADHD. It should be noted that the suggestion that hyperactivity/impulsivity in females may be expressed more through socially oriented behaviors specifically concerns the hyperactivity/impulsivity symptom domain and does not conflict with evidence that females may show greater endorsement of certain task-related inattention symptoms, such as being easily distracted or having difficulty sustaining attention.

However, because most DSM-defined ADHD symptoms appear to function similarly across sexes and sex-related differences have been identified for only a subset of items, the available evidence does not yet justify broad modification of the DSM symptom criteria. Rather, further research may help determine whether targeted refinement of specific items could improve sensitivity to female presentations while preserving symptoms that function comparably across sexes.

### 3.3. DSM-5 Symptom Thresholds

The DSM-5 requires at least six symptoms in either the inattention or hyperactivity/impulsivity domain (or in both) for children and adolescents, with a lower threshold of five symptoms for individuals aged 17 years or older. Critics argue that these cutoffs, originally established in the DSM-IV and carried forward to the DSM-5, reflect symptom patterns more typical of males than females. Consequently, the criteria may fail to recognize that females can experience levels of impairment comparable to males while displaying fewer than six symptoms in either domain, introducing a potential sex bias in diagnosis [16]. Research consistently shows that, particularly in childhood and adolescence, females exhibit fewer overt ADHD symptoms, especially hyperactivity and impulsivity, than their male peers [30]. Achenbach et al. reported similar findings for inattention symptoms and found that, in a clinically referred sample, parents of females endorsed only about half as many inattention items as parents of males on the Achenbach, Conners, and Quay Questionnaire. These empirical findings suggest that, to meet the same DSM criteria, females must show a substantially greater degree of attentional difficulty relative to other girls than males must show relative to other boys. In other words, the existing thresholds require girls to be more deviant from their same-sex peers to qualify for an ADHD diagnosis [31].

Consistent with this concern, more recent population-based data have shown that odds ratios across all symptom domains are slightly higher in females than in males, implying that girls may need to exceed a higher symptom threshold to receive a clinical ADHD diagnosis [3,25]. These findings reinforce earlier concerns raised by researchers in the 1990s, who argued that the existing thresholds may not adequately capture female presentations of ADHD. In fact, these observations led some researchers to propose modified criteria incorporating sex-specific thresholds based on the degree of deviance from sex-referenced norms [32]. However, at a 1994 National Institute of Mental Health (NIMH) conference on sex differences in ADHD, experts concluded that adopting separate sex-normed diagnostic cutoffs was premature. They cautioned that lowering the threshold for females might lead to overdiagnosis and recommended further research before implementing sex-specific norms [30]. Subsequent research supported the use of sex-specific norms for ADHD behavior rating scales and recommended applying separate norms for males and females. For example, scales developed by DuPaul and colleagues for children and adolescents [33] and by Barkley for adults [34] allow females to be compared with same-sex peers when evaluating developmental deviance. Using sex-normed rating scales could help normalize the observed male-to-female ratio in childhood ADHD prevalence [35]. However, this approach might obscure important etiological factors, such as the influence of sex hormones on brain development in ADHD [36], and must be accompanied by evidence that females identified through sex-specific norms exhibit clinically significant impairment [11].

Another approach is to consider individuals with subthreshold symptoms. One study identified a subgroup of girls with subthreshold ADHD symptoms who showed greater functional impairment than girls with lower ADHD scores, although less impairment than females who met the full diagnostic threshold. However, no equivalent subgroup was found among boys. Recognizing such a sex-specific threshold could help identify females who warrant further ADHD assessment [37].

Overall, the current evidence raises legitimate concerns about the sensitivity of fixed symptom thresholds for identifying female presentations of ADHD but does not yet establish that sex-specific diagnostic thresholds would improve diagnostic validity without increasing the risk of false-positive diagnoses. Further research is therefore needed before modifications to the formal DSM symptom thresholds can be justified.

### 3.4. DSM-5 Age-of-Onset Criterion and Late-Onset ADHD

Recent research suggests that the age of ADHD onset differs between sexes, with females more likely to show a marked increase in symptoms during early adolescence, whereas males typically exhibit elevated symptoms from early childhood. Hyperactivity/impulsivity symptoms in females often begin to rise in early adolescence, while in males these elevations are already evident around age 7, although a further increase also occurs during adolescence. Consequently, females who develop hyperactivity/impulsivity later may be excluded from diagnosis because the DSM-5 requires symptom onset before age 12 [38]. The onset of inattentive symptoms also appears to be delayed in females. These symptoms often remain below the diagnostic threshold and are only minimally disruptive in the relatively stable environments of childhood, but they may cross into the clinical range during adolescence, with onset often delayed [39,40].

Growing evidence also challenges the DSM-5 age-12 cutoff by identifying a subgroup often referred to as late-onset ADHD, which includes individuals who show little or no ADHD symptoms in early childhood but develop them in later adolescence or adulthood. Individuals with late-onset ADHD are more likely to be female, whereas those with childhood-onset persistent ADHD show the typical male predominance [41,42,43]. Some female adults with late-onset ADHD appear to have had subtle early-life cognitive difficulties and comparable genetic liability for ADHD [44,45]. However, previous research has suggested that lower cognitive demands in early schooling, attendance at private schools, stronger family support, and the use of compensatory or masking strategies may have prevented these difficulties from causing functional impairment [9]. Symptoms can later emerge or become more impairing during adolescence as academic demands increase, parental support declines, independence grows, and hormonal changes associated with puberty, beginning after menarche, intensify mood and ADHD severity [6,46]. This pattern suggests that at least part of the observed sex bias in ADHD diagnosis can be explained by late or missed diagnosis in females [9]. It also helps account for the narrowing male-to-female ADHD ratio seen in adulthood, where it decreases to roughly 2:1 or even approaches 1:1 in many population samples [29]. Although one might argue that this decline reflects sex differences in the persistence or remission of ADHD symptoms, evidence indicates that males and females are equally likely to experience symptom remission by adulthood [47,48]. Another factor that may contribute to this convergence is the greater reliance on self-reports in adult diagnostic assessments. Because children depend on parents or teachers for referral, girls may remain undiagnosed until they reach adulthood, when increased autonomy allows them to recognize the distress and impairment caused by ADHD and to seek evaluation and support through self-referral [5]. Studies indicate that help-seeking for ADHD is more common among females, even though they are typically diagnosed at a later age, with a mean age of 12.6 years in females and 10.9 years in males [11,49,50]. Taken together, these findings reinforce the view that late or missed diagnosis, along with delayed help-seeking in females, offers the most plausible explanation for the reduced sex discrepancy in adult ADHD prevalence [7].

However, evidence that some individuals identified as having late-onset ADHD had subtle difficulties earlier in life suggests that apparent late onset may sometimes reflect delayed recognition rather than genuinely new symptom onset. Therefore, although the age-12 criterion may contribute to missed or delayed diagnosis in some females, the available evidence does not yet clearly justify modifying or removing the DSM-5 age-of-onset requirement.

### 3.5. Functional Impairment

Research shows that the impact of ADHD symptoms on daily functioning, for example at school, at work, and in relationships, also differs between males and females. In a large population-based study, Mowlem et al. [51] compared girls and boys who met full ADHD diagnostic criteria with those who, despite elevated symptoms, did not reach the diagnostic threshold. The study identified two key findings that may help explain the under-recognition of ADHD in girls. First, girls who met full ADHD criteria had significantly more emotional, conduct, and peer problems than high-symptom girls who were not diagnosed, whereas this contrast was less pronounced for boys. In other words, emotional and behavioral problems were particularly important in distinguishing diagnosed from high-symptom girls but played a smaller role in boys. Consistent with these findings, empirical evidence suggests that girls may need to exhibit a greater burden of co-occurring emotional or behavioral problems and associated functional impairment in order to meet DSM ADHD criteria [52]. These findings also suggest that emotional symptoms may be particularly relevant to the female ADHD phenotype and that girls may express ADHD-related difficulties differently from boys [53,54]. This is important because emotional dysregulation is not explicitly represented among the current DSM diagnostic criteria for ADHD. Second, parents rated boys who met diagnostic criteria as more functionally impaired than boys with high symptom levels who did not meet diagnostic criteria. Among girls, however, parents did not make the same distinction and tended to underestimate hyperactive and impulsive symptoms in girls who met diagnostic criteria. In other words, parent-rated impairment differentiated diagnosed from high-symptom boys but not girls. These sex-related parental perceptions of ADHD impairment suggest that parents may be less able to recognize emotional difficulties in girls [55] and may underestimate their functional impairment, highlighting the need for objective measures of impairment specifically for girls [22]. This may occur because emotional problems are not viewed as problematic as the more disruptive behaviors typically displayed by boys, which usually increase the likelihood of referral [51]. These findings align with evidence that proportionally fewer girls than boys with ADHD annoy or upset their teachers (38% vs. 64%) [56] and that parents perceive “feminine” ADHD symptom items as less problematic than the “masculine” ones [27]. This pattern, often referred to as “recognition bias”, will be discussed in the following section [57].

### 3.6. DSM-5 Pervasiveness Criterion and Informant Recognition

The DSM-5 requires that ADHD symptoms be present in at least two settings, such as at home and at school for children, to meet the pervasiveness criterion. For children and younger adolescents, clinicians typically assess pervasiveness by collecting symptom ratings from at least two informants, usually parents and teachers. First, in a population-based study, parent–teacher ratings were less strongly correlated for females than for males in early adolescence [58]. This weaker agreement supports the idea that females are more likely than males to display situational ADHD, with symptoms that appear more prominently in one setting, particularly at home [59]. Second, the male-to-female ratio for ADHD prevalence is higher when parent–teacher ratings concur on the presence of ADHD (5.3:1) compared with teacher-only ratings (3.3:1) or parent-only ratings (2.5:1) [60]. These ratios indicate that females are less likely than males to meet the symptom pervasiveness criterion when concordant parent–teacher reports are required. However, this pattern does not necessarily reflect true situational differences in symptom expression alone and may also be influenced by sex-related differences in informant perception, particularly teacher perception, and by the reference groups against which children’s behaviors are judged.

One proposed explanation for these findings is the nature of the symptoms typically observed in females with ADHD. Females more often exhibit inattentive symptoms (e.g., daydreaming, restless thoughts, and mind-wandering) [16]. These symptoms are less outwardly disruptive and therefore more difficult for informants, especially teachers, to detect than the externalizing, disruptive, impulsive, and hyperactive behaviors that are more common in males [61]. Supporting this view, females are more likely than males to be diagnosed with the ADHD-PI presentation [62] and less likely to be diagnosed with the ADHD-C presentation [17]. This difference in symptom presentation may help explain the observed sex differences in informant agreement and the lower likelihood that females meet the DSM pervasiveness criterion. Based on previous literature, two potential approaches have been proposed to enhance diagnostic accuracy. First, self-reported symptom ratings may better capture the more internal and inattentive presentations that are more common in females than in males [40]. Second, refining the pervasiveness criterion itself could improve the accuracy of ADHD assessment in females [6]. Nevertheless, it is important to note that requiring evidence of symptoms across multiple settings serves an important diagnostic function by helping distinguish pervasive ADHD-related difficulties from problems that are primarily context-specific. Moreover, lower agreement between informants does not necessarily indicate that the pervasiveness criterion itself is invalid, as it may partly reflect genuine variation in symptom expression across settings or differences in informant perception. Therefore, the available evidence supports careful interpretation of multi-informant assessments in females but does not yet justify removing or substantially modifying the DSM-5 pervasiveness criterion.

A further contributing factor is the way the DSM diagnostic framework has shaped how key informants perceive ADHD. Over time, the male-biased DSM criteria appear to have influenced how parents and teachers, who play a central role in referral and diagnosis, recognize ADHD-related behaviors. For example, parents have reported that the DSM-IV ADHD criteria seem more descriptive of boys [27]. This prevailing perception, combined with common sex-based expectations of children’s behavior, has reinforced the stereotype of ADHD as a “disruptive boy” disorder. Such assumptions create what is often termed “recognition bias” and can hinder the identification of ADHD in girls, contributing to their underdiagnosis [51,63].

### 3.7. Diagnostic Overshadowing

The emotional difficulties experienced by females with ADHD have been proposed to be particularly important to the female ADHD phenotype and may represent a distinct way in which impairment manifests [51]. These emotional difficulties may overshadow their ADHD symptoms by taking the form of internalizing problems such as anxiety and depression, which in turn may lead clinicians to assign alternative diagnoses rather than recognize ADHD [35,64]. Similarly, in adult females, ADHD has been found to be more strongly associated with anxiety, depression, bipolar disorder, and personality disorders compared with adult males [65]. Such diagnostic overshadowing may delay the diagnosis of ADHD in females and reduce timely access to appropriate clinical care and treatment [26].

Hyperactivity and impulsivity in females have also been proposed to manifest as externalizing verbal behaviors that may be misinterpreted as symptoms of anxiety disorders, borderline personality disorder, or bipolar disorder [9,66,67]. This misinterpretation may further explain why females are more often directed toward therapeutic psychological interventions, whereas males are more likely to be referred for medical ADHD assessment and treatment [68,69].

### 3.8. Biological and Developmental Contributions to Sex-Related Diagnostic Bias in ADHD

Biological and developmental factors may also contribute to sex differences in the timing and expression of ADHD symptoms, which may in turn interact with diagnostic processes. Developmental trajectories appear to differ between males and females, with ADHD symptoms generally becoming apparent earlier in boys, whereas symptoms in girls may become more prominent or impairing during adolescence [7]. Pubertal hormonal changes may contribute to this divergence. Sex-related hormonal influences have been linked to developmental differences in dopaminergic systems [4,70], while rising estrogen levels during puberty have been associated with changes in striatal dopamine D2 receptor availability in females [71]. These developmental differences may have diagnostic implications because criteria, referral practices, and clinical expectations have historically been shaped by the more overt childhood presentations commonly observed in males. Consequently, females whose symptoms emerge, intensify, or become functionally impairing later in development may be less readily recognized.

ADHD-related functioning and symptom expression in females may also vary across reproductive hormonal states. Increasing attention has been directed toward the potential influence of hormonal changes during puberty, across the menstrual cycle, during pregnancy and the postpartum period, and through perimenopause and menopause in women with ADHD [72]. However, research examining these female-specific hormonal influences remains limited. Fluctuations in estrogen have been linked to changes in emotion regulation and hormone-related mood disturbances in females with ADHD, including difficulties associated with the premenstrual period and other reproductive transitions [73]. Hormonal changes associated with pregnancy, postpartum periods, and later reproductive transitions may also interact with ADHD symptom expression and functional impairment across the female lifespan [74,75]. Such variability may further complicate clinical recognition by contributing to variability in symptom expression and functioning across developmental and reproductive stages. Importantly, these biological and developmental influences are not themselves sources of diagnostic bias; rather, they may shape ADHD presentations in ways that interact with diagnostic expectations, recognition, and assessment practices.

## 4. Sex-Related Diagnostic Bias in Adulthood

Although sex-related disparities in ADHD recognition are particularly evident during childhood, diagnostic bias can persist into adulthood and create additional challenges for individuals and healthcare systems. A large Swedish register study found that females received an ADHD diagnosis approximately four years later than males despite substantial prior contact with healthcare services [76]. These findings suggest that women may reach adulthood after years of unrecognized ADHD symptoms or treatment for other psychiatric difficulties. Several factors may contribute to this delayed recognition in adulthood.

First, mood and anxiety symptoms may complicate the recognition of ADHD in adult females through two potentially overlapping pathways. Inattention, executive-function difficulties, and emotional symptoms may be more prominent than overt hyperactivity in females, while co-occurring anxiety and depressive disorders may obscure underlying ADHD and lead to alternative diagnoses. In one pathway, mood or anxiety symptoms may develop in the context of longstanding difficulties and functional impairment associated with unrecognized ADHD and subsequently become the primary focus of clinical attention. A recent registry-based study in Wales found that women prescribed an antidepressant before their ADHD diagnosis were more likely than men to discontinue it after receiving an ADHD diagnosis [50]. This pattern has been interpreted as suggesting that, in some women, mood or anxiety symptoms may develop secondary to unrecognized ADHD, whereas in other cases the resulting diagnosis may represent misdiagnosis [72]. However, this should be interpreted cautiously, as mood and anxiety disorders commonly co-occur with ADHD; thus, earlier diagnoses may also reflect genuine comorbidity rather than misdiagnosis. In another pathway, some females may develop compensatory or masking strategies that reduce the visibility of their ADHD symptoms and delay recognition. These sustained compensatory efforts have also been suggested to contribute to psychological stress and anxiety [72].

Second, adult assessment also presents a distinct diagnostic challenge because the DSM-5 requires evidence of symptoms beginning in childhood. Adults seeking a first ADHD diagnosis may have difficulty accurately recalling childhood symptoms, and retrospective reports from patients or other informants may be affected by limitations of long-term memory [77]. This problem may be particularly relevant to women whose childhood symptoms were less disruptive or were masked or compensated for and therefore may have gone unrecognized, making retrospective confirmation of childhood symptom onset more difficult later in life [78].

## 5. Clinical Implications

Because sex-related diagnostic biases may contribute to missed or delayed recognition of ADHD in females, these biases have important clinical implications [76]. Women who remain undiagnosed until adulthood have been reported to experience persistent difficulties in self-esteem, social-emotional well-being, and interpersonal relationships [79]. Delayed recognition may also leave educational and occupational difficulties insufficiently addressed during important developmental periods and postpone access to appropriate ADHD treatment and support [40,79]. Delayed or missed diagnosis may also prolong ADHD-related impairment and psychological distress, potentially contributing to a greater long-term psychiatric burden, including anxiety and depressive symptoms. In addition, females with ADHD may experience higher healthcare utilization before receiving an ADHD diagnosis [76].

Earlier and more accurate identification may help reduce the duration of untreated ADHD-related impairment and provide females with earlier access to evidence-based interventions. Improving the recognition of ADHD in females will likely require greater awareness among parents, educators, and healthcare providers of the heterogeneity of ADHD presentations beyond the traditional hyperactive-disruptive profile. Incorporating self-report measures, systematic assessment of functional impairment, and greater consideration of internalizing symptoms may enhance diagnostic accuracy [40]. Ultimately, a more nuanced understanding of sex-related differences and diagnostic biases will be essential for ensuring equitable identification, assessment, and treatment of ADHD across sexes.

## 6. Sex, Gender, and Sociocultural Influences on Diagnostic Bias

Biological sex is typically identified as a binary characteristic (female or male) assigned at birth, encompassing chromosomal, genetic, gonadal, hormonal, and other anatomical or physiological traits, although a subgroup of intersex individuals does not fit neatly into this binary. Gender identity, by contrast, refers to a person’s internal sense of being feminine (e.g., a girl or woman), masculine (e.g., a boy or man), or another identity outside the binary (e.g., non-binary), and it can vary across cultures and change over time [12]. Biological sex may contribute to differences in neurodevelopmental trajectories, hormonal influences, and symptom expression, whereas gender-related expectations and behavioral norms may shape how similar symptoms are expressed, perceived, and reported. Sociocultural factors may further influence whether particular behaviors are regarded as problematic, how parents and teachers recognize ADHD symptoms, and which individuals are referred for clinical assessment.

Although biological sex and gender are conceptually distinct, most ADHD research, especially studies of children and adolescents, often uses these terms interchangeably. Because most existing studies do not directly measure gender identity, gender expression, or sociocultural expectations separately from biological sex, the relative contribution of these factors cannot yet be clearly disentangled [13]. Thus, some differences described as “sex differences” in the literature may reflect a combination of biological and socially mediated processes [6]. Taken together, variations in the clinical presentation and developmental expression of ADHD, as well as observed male-female differences in ADHD recognition and diagnosis, likely reflect an interplay between biological sex, gender-related factors, and broader sociocultural influences rather than any single mechanism.

## 7. Limitations and Generalizability Considerations

Several limitations of the existing literature on sex-related differences and diagnostic bias in ADHD should be considered when interpreting the evidence reviewed here. Relatively few studies have specifically prioritized the examination of sex differences in ADHD, and reported differences have generally been modest in magnitude [38]. Much of the available research is observational and often cross-sectional, limiting causal conclusions regarding whether reported sex differences reflect diagnostic bias, underlying differences in ADHD presentation, or both. Publication bias may also be present, as studies reporting significant sex-related differences may be more likely to be published than studies reporting null findings, potentially leading to an overestimation of some effects. In addition, studies vary considerably in the diagnostic criteria and assessment instruments used, including differences in DSM versions, symptom-rating scales, informant sources, and thresholds for meeting criteria for a clinical ADHD diagnosis. Considerable heterogeneity is also present across study populations with respect to age, clinical versus community sampling, and demographic characteristics [11]. These methodological differences may contribute to inconsistencies across studies and limit the generalizability of some findings.

The generalizability of the findings reviewed here to different healthcare systems and cultural contexts should also be considered. Cultural perceptions of ADHD symptoms, gender-related expectations, professional and public awareness, access to specialist assessment, and the organization of referral and mental health services may all vary across settings and influence the recognition of ADHD and the magnitude of observed sex-related diagnostic disparities [40]. For example, one study investigated whether parents in the UK and Hong Kong use different ADHD symptom endorsement thresholds and found that, despite lower objectively measured activity levels among children in Hong Kong, parents in Hong Kong reported higher levels of hyperactivity/impulsivity and inattention than parents in the United Kingdom. These findings suggest that cultural context may influence the threshold at which ADHD-related behaviors are perceived and endorsed as clinically relevant symptoms [80]. Gender-related expectations may further shape how ADHD-related behaviors are perceived by parents and educators and whether such behaviors prompt help-seeking or referral [81]. Healthcare and referral structures may also differ across countries, shaping barriers to ADHD assessment [82]. For example, a recent UK longitudinal study found that parents were frequently the instigators of the ADHD diagnostic process and often had to navigate prolonged referral and assessment pathways, advocate for their concerns to be recognized, and overcome barriers within healthcare and educational systems to access assessment and support [83].

## 8. Conclusions and Future Directions

**Figure 1:**
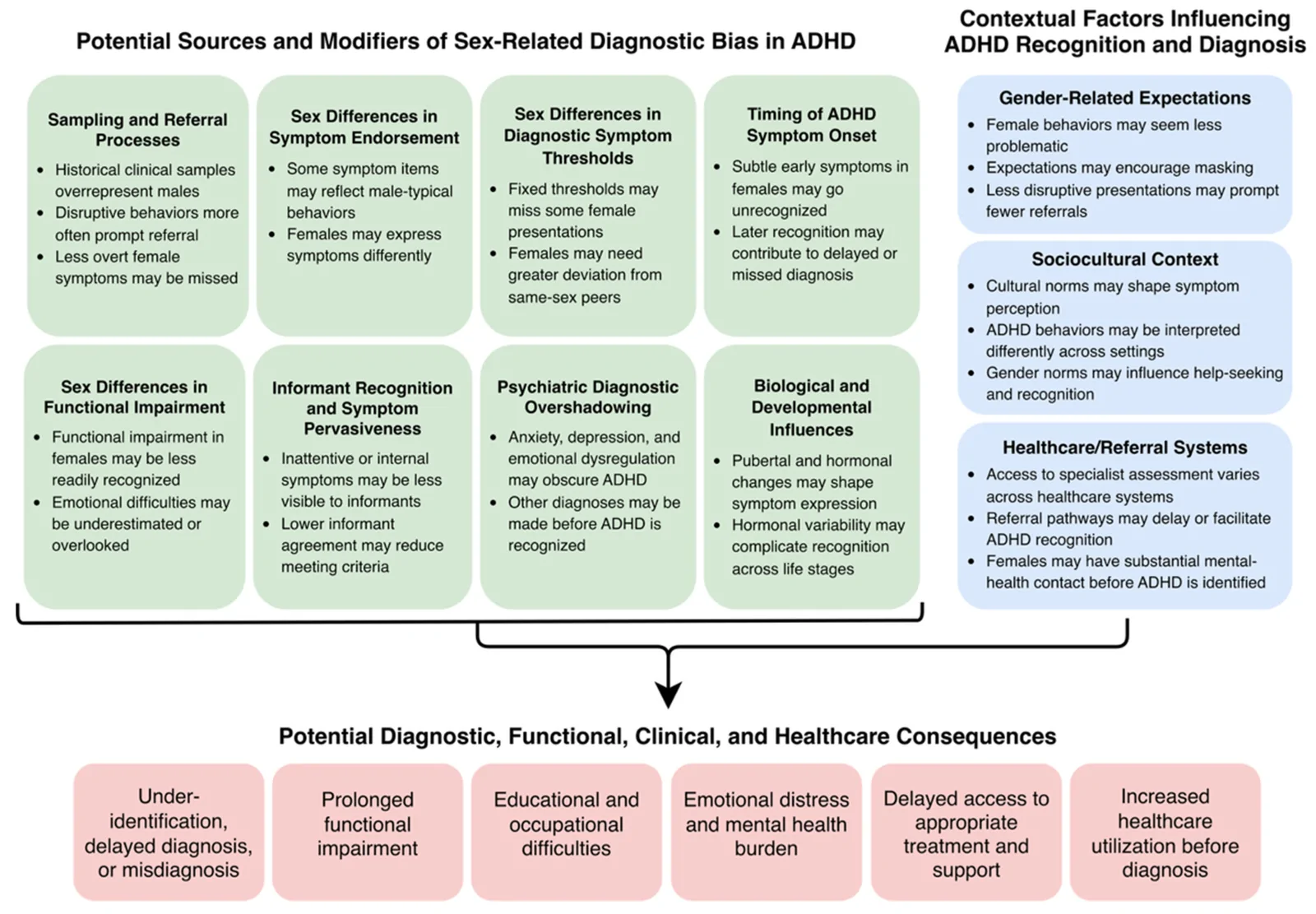
Conceptual framework of potential sources, modifiers, contextual influences, and consequences of sex-related diagnostic bias in ADHD.

The longstanding perception of ADHD as a predominantly male disorder has shaped both scientific understanding and clinical practice for decades. Although accumulating evidence supports the existence of genuine sex-related differences in symptom expression, developmental trajectories, and neurobiological mechanisms, the findings reviewed here suggest that methodological, diagnostic, and sociocultural factors may also contribute to the observed male-female disparity in ADHD diagnosis and may collectively reduce the likelihood that females with ADHD are accurately identified and diagnosed. Biological and developmental factors, including hormonal influences across developmental and reproductive stages, may further shape the timing and expression of ADHD symptoms and interact with diagnostic recognition and assessment. A conceptual overview of the principal sources and modifiers of sex-related diagnostic bias, relevant contextual influences, and their potential diagnostic and downstream consequences is presented in Figure 1.

In adulthood, sex-related diagnostic bias may reflect both the persistence of missed recognition from childhood and adult-specific assessment challenges, including diagnostic overshadowing by psychiatric comorbidity, retrospective verification of childhood symptom onset, and less overt or masked/compensated ADHD presentations.

The magnitude and mechanisms of sex-related disparities in ADHD recognition and diagnosis may also vary across cultural and healthcare contexts, as differences in symptom perception, gender-related expectations, referral pathways, and access to specialist assessment may shape the likelihood of ADHD recognition and diagnosis.

Future research should prioritize large-scale longitudinal studies with adequate female representation across developmental stages, from childhood through adulthood. Greater attention should be devoted to understanding how ADHD symptoms manifest in females and whether current diagnostic criteria adequately capture these presentations. Adult-focused research should further examine the factors contributing to delayed diagnosis in women and the reliability of retrospective assessment of childhood symptom onset. Studies examining differential item functioning, sex-specific developmental trajectories, and the clinical utility of sex-referenced norms may help determine whether modifications to existing diagnostic frameworks are warranted. Further research is also needed to evaluate the role of emotional dysregulation, compensatory coping strategies, masking behaviors, and hormonal influences in shaping ADHD presentations in females. Addressing these gaps may help distinguish genuine sex-related variation in ADHD presentation from diagnostic bias and support more equitable identification and clinical care across sexes.

## Data Availability

The original contributions presented in this study are included in the article. Further inquiries can be directed to the corresponding author.
